# Association between chemerin, omentin-1 and risk of heart failure in the population-based EPIC-Potsdam study

**DOI:** 10.1038/s41598-017-14518-2

**Published:** 2017-10-26

**Authors:** Juliane Menzel, Romina di Giuseppe, Ronald Biemann, Clemens Wittenbecher, Krasimira Aleksandrova, Fabian Eichelmann, Andreas Fritsche, Matthias B. Schulze, Heiner Boeing, Berend Isermann, Cornelia Weikert

**Affiliations:** 1 Department of Molecular Epidemiology, German Institute of Human Nutrition Potsdam–Rehbruecke, Nuthetal, Germany; 2 German Federal Institute for Risk Assessment, Department of Food Safety, Berlin, Germany; 3 Institute for Social Medicine, Epidemiology and Health Economics, Charité University Medical Center, Berlin, Germany; 4 German Center for Diabetes Research (DZD), München-Neuherberg, Germany; 5 Institute of Epidemiology, Christian-Albrechts University Kiel, Kiel, Germany; 6 Institute for Clinical Chemistry and Pathobiochemistry, Otto-von-Guericke University Magdeburg, Magdeburg, Germany; 7 Nutrition, Immunity and Metabolism Start-up Lab, Department of Epidemiology, German Institute of Human Nutrition Potsdam-Rehbruecke, Nuthetal, Germany; 8 Department of Epidemiology, German Institute of Human Nutrition Potsdam-Rehbruecke, Nuthetal, Germany; 9 Department of Internal Medicine, Division of Endocrinology, Diabetology, Nephrology, Vascular Disease and Clinical Chemistry, University of Tübingen, Tübingen, Germany

## Abstract

The adipokines chemerin and omentin-1 have been suggested to influence cardiovascular function. The study aimed to investigate the longitudinal association between chemerin, omentin-1 concentrations and risk of incident heart failure (HF), respectively. We conducted a case-cohort study nested within the European Prospective Investigation into Cancer and Nutrition (EPIC)-Potsdam cohort (n = 27548) including a randomly drawn subsample and all incident HF cases during a mean follow-up of 8.2 ± 1.5 years. A total of 212 incident HF cases and 2168 individuals free of HF cases were included in the study. After multivariable adjustment for established cardiovascular risk factors chemerin was strongly associated with risk of HF (HR per doubling chemerin: 4.91; 95%-CI: 2.57–9.39; p < 0.0001). Omentin-1 was not significantly related to HF risk in the overall study population. However, the association between omentin-1 and HF risk was modified by prevalent coronary heart disease (CHD), showing that the shape of the association was linear in participants without prevalent CHD (HR doubling omentin-1: 2.11; 95%-CI: 1.36–3.27; p linear = 0.0009) and U-shaped in participants with pre-existing CHD (p non-linear = 0.006). Our study provides first evidence for a strong positive association between chemerin and risk of HF. The association between the adipokine omentin-1 and risk of HF may differ according to pre-existing CHD.

## Introduction

Heart failure (HF) is a complex syndrome with growing public health burden, characterized by frequent hospitalization and reduced quality of life^1^. In spite of improved therapeutic treatments, HF is still a leading cause of death globally^2^. The pathophysiology of HF is complex, however independent risk factors have been identified, foremost coronary heart disease (CHD), as well as hypertension, diabetes and obesity^2,3^. The discovery of adipokines identified adipose tissue as an important key factor in the organ crosstalk network^4^, including cardiovascular function^5,6^. Nowadays, it has been suggested that chemerin and omentin-1 may be suitable candidates to influence cardiovascular health.

Chemerin, also known as TIG2 (tazarotene-induced gene 2) or RARRES2 (retinoic acid receptor responder protein 2) is mainly produced in adipose tissue and in the liver^7^. It has been shown that circulating chemerin concentrations were associated with the higher presence of coronary artery disease (CAD)^8–12^ and severity of coronary atherosclerosis^8,9,12^. Moreover, high chemerin concentrations were independently associated with arterial stiffness^13^, and with carotid artery plaque instability^14^. Recently, Leiherer et al. observed an association between higher chemerin concentrations and higher risk of further cardiovascular events in patients with established or suspected stable CAD in a prospective study^15^. Indeed, scientific evidence proposed chemerin as a biomarker with adverse effects on cardiovascular health, however less is known about chemerin and risk of HF.

Omentin-1 is a novel adipokine primarily released from visceral adipose tissue^16^. So far, conflicting results were reported from studies investigating the association between omentin-1 and different cardiovascular endpoints. On the one hand studies promoted omentin-1 as cardio-protective adipokine, showing cross-sectional associations of omentin-1 with several cardiometabolic parameters e.g. inverse association between omentin-1 and carotid artery intima-media thickness in patients with metabolic syndrome^17^ and decreased omentin-1 associated with cardiovascular dysfunction in patients with type 2 diabetes^18^. On the other hand prospective studies suggested omentin-1 as cardiovascular risk factor^19,20^ e.g. our previous study performed in the European Prospective Investigation into Cancer and Nutrition (EPIC)-Potsdam study showed that higher omentin-1 concentrations were associated with a higher risk of stroke, particular in metabolically healthy participants (e.g. normal waist circumference, low C-reactive protein concentrations)^19^. Interestingly, a recently published study noticed that higher omentin-1 concentrations were related to increased left ventricular volumes and dysfunction in patients with chronic HF^16,21^. The latter may in particular suggest an involvement of omentin-1 in the pathogenesis of HF.

Up to now, studies investigating the relationship between chemerin, omentin-1 and HF incidence are still missing. Therefore, the present prospective study aimed to investigate the longitudinal association between circulating chemerin and omentin-1 concentrations and the risk of HF in the population-based EPIC-Potsdam study. Further we aimed to investigate possible effect modifications of important risk factors for HF i.e. sex, obesity, inflammation and the prevalent diseases diabetes, hypertension and CHD.

## Results

### General and biochemical characteristics

Medians and interquartile ranges of chemerin and omentin-1 were 147.3 ng/ml (125.3–170.5) and 397.8 ng/ml (327.7–489.5) in the subcohort and 180.0 ng/ml (156.1–206.2) and 452.6 ng/ml (368.9–569.7) in HF cases. The distribution (sex- and age-adjusted mean (95%-CI) or percentage) of general and biochemical baseline characteristics according to quartiles of chemerin and omentin-1 across the subcohort are shown in Table 1 and Table 2. Participants with higher chemerin concentrations have higher waist circumference (1. quartile (Q1): 81.8 (80.9–82.6) cm vs. 4. quartile (Q4): 93.6 (92.7–94.4) cm; p for trend < 0.0001), and were more likely to suffer from prevalent diabetes (Q1: 3.8% vs. Q4: 7.6%; p for trend = 0.003), metabolic syndrome (Q1: 29.8% vs. Q4: 64.4%; p for trend < 0.0001), or hypertension (Q1: 38.3% vs. Q4: 63.4%; p for trend < 0.0001). The blood-based biomarker concentrations of total cholesterol, triglycerides and high sensitivity C-reactive protein (hsCRP) were on average higher in participants with higher chemerin level (all p for trend < 0.0001). Moreover, we observed inverse associations across quartiles of chemerin with high density lipoprotein (HDL)-cholesterol and omentin-1 (all p for trend < 0.05) (Table 1). In contrast, participants with higher omentin-1 concentrations have lower waist circumference (Q1: 89.8 (88.9–90.7) cm vs. Q4: 85.2 (84.3–86.1) cm; p for trend < 0.0001) and were more likely to have a history of prevalent CHD (Q1: 8.0% vs. Q4: 11.1%; p for trend = 0.04). Further, omentin-1 showed a significant positive association with HDL-cholesterol, a significant inverse association was observed with hsCRP (all p for trend < 0.05) (Table 2). Furthermore, in Supplementary Table S1 Spearman sex- and age-adjusted partial correlation coefficients on the associations of chemerin and omentin-1 in relation to general and biochemical baseline characteristics are presented, and in Supplementary Table S2 the clinical characteristics of HF cases are summarized.

**Table 1:** General and biochemical characteristics according to quartiles of chemerin within the subcohort (n = 2190) of EPIC-Potsdam study.

Characteristics	Quartiles of chemerin in the subcohort^a^				P for trend
	Q1	Q2	Q3	Q4	
n	545	550	548	547	
Chemerin [ng/ml]^b^	111.7 (101.8–119.3)	136.9 (131.1–141.6)	157.8 (152.0–163.6)	191.9 (180.2–208.8)	
Men [%]^c^	42.2	40.8	38.7	33.5	0.003
Age [years]^c^	47.1 (46.4–47.9)	50.6 (49.9–51.3)	51.2 (50.4–51.9)	54.0 (53.3–54.8)	<0.0001
Waist circumference [cm]	81.8 (80.9–82.6)	85.7 (84.8–86.5)	88.6 (87.8–89.4)	93.6 (92.7–94.4)	<0.0001
Physical activity [h/week]	1.3 (1.2–1.5)	1.0 (0.8–1.1)	1.0 (0.8–1.1)	0.8 (0.7–1.0)	<0.0001
Smoking [%]					
Non-Smoker	47.4	44.4	41.7	40.0	0.008
Ex-Smoker	34.4	34.8	34.6	35.5	0.72
Smoker < 20 cigarettes/day	13.4	14.6	16.1	16.8	0.10
Smoker ≥ 20 cigarettes/day	4.8	6.1	7.7	7.7	0.02
Education [%]					
Unskilled or skilled	32.7	35.6	36.8	39.1	0.03
Technical College	22.7	21.4	24.6	22.8	0.70
University degree	44.5	43.1	38.6	38.1	0.01
Alcohol consumption [%]					
Never	0.6	0.7	0.1	0.1	0.13
Ex-Drinker	3.0	3.0	2.2	2.0	0.24
Current (≤12 g women /≤24 g men)	65.2	68.6	68.0	71.9	0.03
Current (>12 g women />24 g men)	31.2	27.7	29.6	26.0	0.11
Prevalent diabetes [%]	3.8	3.4	4.4	7.6	0.003
Prevalent hypertension [%]	38.3	47.7	53.6	63.4	<0.0001
Prevalent CHD [%]	7.1	9.1	7.7	10.2	0.13
Prevalent metabolic syndrome [%]^d^	29.8	42.0	50.4	64.4	<0.0001
Total cholesterol [mmol/l]	5.08 (4.99–5.17)	5.20 (5.11–5.29)	5.34 (5.25–5.43)	5.55 (5.46–5.64)	<0.0001
HDL-cholesterol [mmol/l]	1.50 (1.47–1.54)	1.45 (1.42–1.48)	1.38 (1.35–1.41)	1.34 (1.30–1.37)	<0.0001
Triglyceride [mmol/l]	1.17 (1.09–1.26)	1.46 (1.38–1.54)	1.68 (1.60–1.76)	2.03 (1.94–2.11)	<0.0001
hsCRP [mg/l]	0.70 (0.41–0.98)	1.47 (1.19–1.75)	2.20 (1.92–2.48)	3.60 (3.31–3.88)	<0.0001
Adiponectin [μg/ml]^e^	9.02 (8.69–9.35)	8.46 (8.14–8.78)	7.71 (7.39–8.03)	7.25 (6.92–7.58)	<0.0001
Omentin-1 [ng/ml]	436.3 (424.7–447.9)	414.7 (403.4–426.1)	417.7 (406.3–429.1)	414.8 (403.1–426.4)	0.02

^a^All variables were adjusted for sex and age; expressed as adjusted percentage or mean and 95%-CI.

^b^Unadjusted variable, expressed as median (interquartile range).

^c^Adjusted for sex, age; according to examined variable.

^d^n = 2153.

^e^n = 2188.

**Table 2:** General and biochemical characteristics according to quartiles of omentin-1 within the subcohort (n = 2190) of EPIC-Potsdam study.

**Characteristics**	**Quartiles of omentin-1 in the subcohort** ^**a**^				**P for trend**
	**Q1**	**Q2**	**Q3**	**Q4**	
n	547	549	548	546	
Omentin-1 [ng/ml]^b^	286.5 (250.6–308.9)	365.4 (346.3–381.6)	441.8 (422.2–465.2)	574.2 (523.0–643.5)	
Men [%] ^c^	42.0	39.7	37.6	35.9	0.03
Age [years]^c^	47.6 (46.9–48.3)	49.1 (48.4–49.8)	52.1 (51.4–52.8)	54.2 (53.5–54.9)	<0.0001
Waist circumference [cm]	89.8 (88.9–90.7)	87.4 (86.5–88.3)	86.8 (85.9–87.7)	85.2 (84.3–86.1)	<0.0001
Physical activity [h/week]	0.91 (0.76–1.05)	0.92 (0.78–1.07)	0.97 (0.82–1.11)	1.29 (1.14–1.43)	0.0006
Smoking [%]					
Non-Smoker	43.2	42.7	40.8	47.0	0.32
Ex-Smoker	34.3	34.1	37.3	33.6	0.90
Smoker < 20 cigarettes/day	15.7	15.5	16.9	12.7	0.28
Smoker ≥ 20 cigarettes/day	6.8	7.7	5.1	6.7	0.52
Education [%]					
Unskilled or skilled	35.8	35.6	36.4	36.4	0.79
Technical College	23.0	23.6	24.1	20.7	0.44
University degree	41.2	40.8	39.5	43.0	0.67
Alcohol consumption [%]					
Never	0.4	0.3	0.3	0.6	0.77
Ex-Drinker	2.1	2.9	2.7	2.4	0.87
Current (≤12 g women/≤24 g men)	72.0	68.3	69.5	63.8	0.009
Current (>12 g women />24 g men)	25.4	28.5	27.5	33.3	0.01
Prevalent diabetes [%]	4.6	3.9	4.6	6.2	0.19
Prevalent hypertension [%]	52.3	50.0	48.9	51.2	0.63
Prevalent CHD [%]	8.0	6.7	8.3	11.1	0.04
Prevalent metabolic syndrome [%]^d^	50.2	46.2	44.5	44.9	0.06
Total cholesterol [mmol/l]	5.23 (5.14–5.32)	5.30 (5.21–5.39)	5.28 (5.19–5.37)	5.35 (5.26–5.45)	0.09
HDL-cholesterol [mmol/l]	1.35 (1.32–1.38)	1.39 (1.36–1.42)	1.41 (1.38–1.44)	1.52 (1.49–1.55)	<0.0001
Triglyceride [mmol/l]	1.63 (1.54–1.72)	1.54 (1.46–1.63)	1.67 (1.58–1.75)	1.48 (1.39–1.57)	0.10
hsCRP [mg/l]	2.54 (2.25–2.83)	1.69 (1.40–1.98)	1.95 (1.66–2.25)	1.70 (1.41–2.00)	0.001
Adiponectin [μg/ml]^e^	7.00 (6.68–7.32)	7.78 (7.45–8.10)	8.28 (7.96–8.60)	9.44 (9.11–9.77)	<0.0001
Chemerin [ng/ml]	152.8 (149.9–155.7)	146.7 (143.9–149.6)	152.4 (149.5–155.3)	148.1 (145.1–151.0)	0.21

^a^All variables were adjusted for sex and age; expressed as adjusted percentage or mean and 95%-CI.

^b^Unadjusted variable, expressed as median (interquartile range).

^c^Adjusted for sex, age; according to examined variable.

^d^n = 2153.

^e^n = 2188.

### Chemerin and the risk of HF

After adjustment for age and sex participants in the highest quartile of chemerin concentrations had a higher risk of HF (Model 1, HR: 9.47; 95%-confidence interval (CI): 4.67–19.2; p linear trend < 0.0001) compared to participants in the lowest quartile (Table 3). After additionally adjustment for waist circumference, physical activity, education, smoking, alcohol consumption, prevalent hypertension, diabetes, CHD, HDL-cholesterol, total cholesterol, triglycerides and hsCRP (Model 4) the association was attenuated, but a strong and significant association remained (HR: 4.23; 95%-CI: 1.92–9.35 for the highest quartile compared to the lowest quartile, p linear trend < 0.0001, Table 3). There was no evidence of departure from linearity for the relation between chemerin and HF risk (p for nonlinearity = 0.41). Interaction analyses revealed no differences in the association between chemerin and risk of HF with respect to sex (p for interaction = 0.97), waist circumference (p for interaction = 0.60), prevalent CHD (p for interaction = 0.36), prevalent diabetes (p for interaction = 0.89), prevalent hypertension (p for interaction = 0.20) or hsCRP (p for interaction = 0.98).

**Table 3:** Hazard ratios of HF according to quartiles of chemerin.

	Quartiles of chemerin levels				*p for trend*	Per doubling of chemerin	
	Q1	Q2	Q3	Q4			*p-value*
Chemerin [ng/ml]^a^	111.7 (101.8–119.3)	136.9 (131.1–141.6)	157.8 (152.0–163.6)	191.9 (180.2–208.8)			
Subcohort participants (n)	545	550	548	547			
Heart failure cases (n)	9	24	52	127			
Model 1^b^	Reference	2.14 (0.97–4.72)	4.27 (2.06–8.84)	9.47 (4.67–19.2)	<0.0001	10.4 (6.39–16.8)	<0.0001
Model 2^c^	Reference	1.77 (0.78–3.99)	3.23 (1.49–7.02)	6.43 (3.02–13.7)	<0.0001	7.20 (4.20–12.3)	<0.0001
Model 3^d^	Reference	1.66 (0.74–3.75)	3.33 (1.53–7.25)	6.27 (2.96–13.3)	<0.0001	6.39 (3.65–11.2)	<0.0001
Model 4^e^	Reference	1.50 (0.65–3.44)	2.49 (1.12–5.57)	4.23 (1.92–9.35)	<0.0001	4.91 (2.57–9.39)	<0.0001

Hazard ratios and 95%-CI were derived from Cox proportional hazard regression.

^a^Quartiles are based on the distribution of chemerin within the subcohort expressed as median and interquartile range.

^b^Model 1: adjusted for age and sex.

^c^Model 2: additionally adjusted for waist circumference, physical activity, education, smoking, alcohol consumption.

^d^Model 3: additionally adjusted prevalent hypertension, diabetes, CHD.

^e^Model 4: additionally adjusted HDL-cholesterol, total cholesterol, triglycerides, hsCRP.

### Omentin-1 and the risk of HF

**Figure 1:**
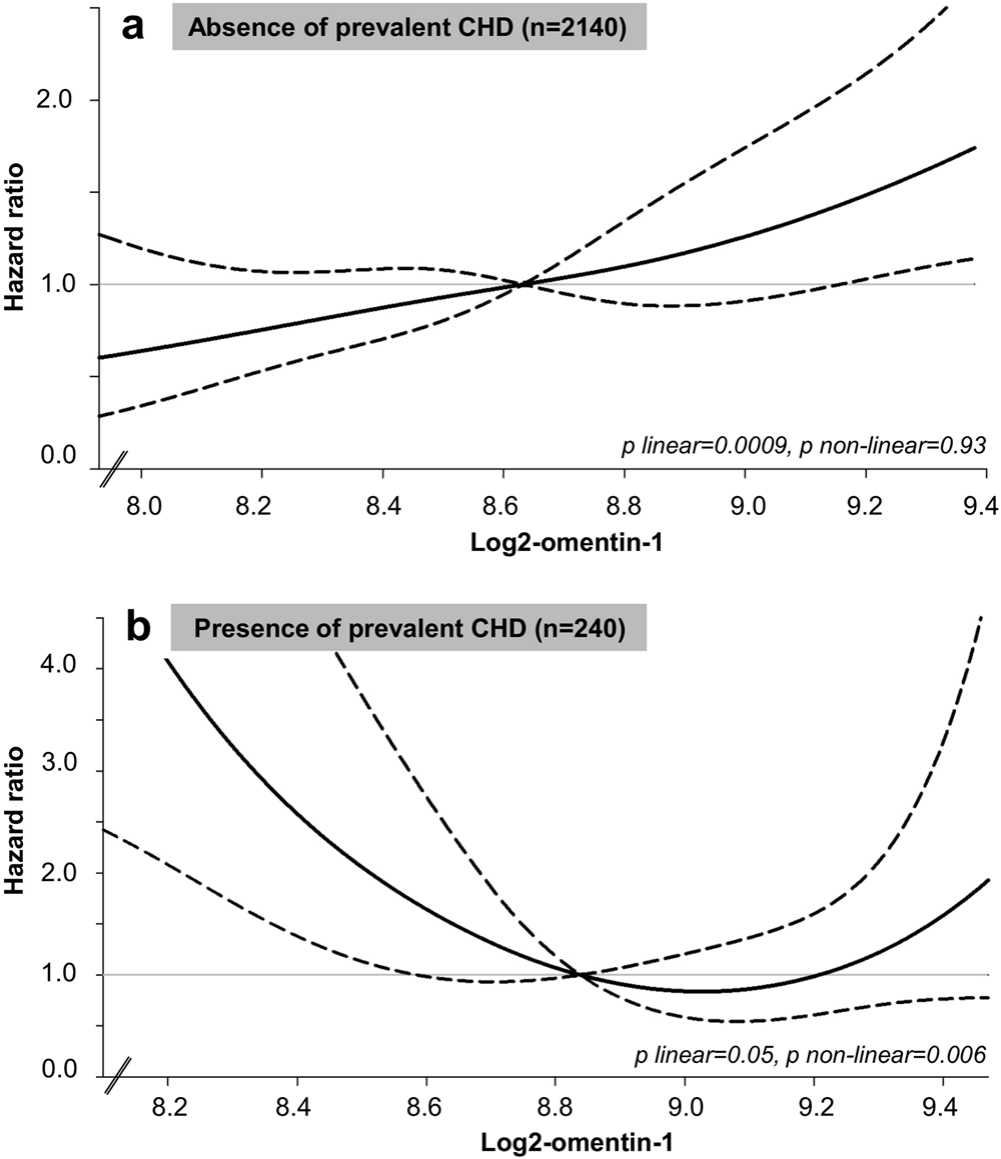
Hazard rate ratio curves for the association between omentin-1 concentrations and the risk of heart failure. (**a**) In participants without prevalent coronary heart disease (n = 2140). (**b**) In participants with prevalent coronary heart disease (n = 240). The solid lines indicate HR of HF as obtained by restricted cubic spline Cox regression with knots placed at fixed values (5^th^, 35^th^, 50^th^ (reference), 65^th^, and 95^th^ percentile of omentin-1 in the entire case-cohort). Dashed lines indicate 95%-CI. Adjusted for age, sex, waist circumference, physical activity, education, smoking, alcohol consumption, prevalent hypertension, diabetes, HDL-cholesterol, total cholesterol, triglycerides, hsCRP. P for non-linearity was calculated by Wald chi-square test.

The present study observed no association between omentin-1 and the risk of HF in the overall study population (Model 4, HR per doubling omentin-1:1.25; 95%-CI: 0.80–1.96, p = 0.32) (Supplementary Table S3). However, the association between omentin-1 and HF risk was modified by prevalent CHD (p for interaction = 0.0005) (Fig. 1). Stratified analyses, conducted in the fully adjusted Model 4, revealed a positive linear association of omentin-1 with the risk of HF (HR doubling omentin-1: 2.11; 95%-CI: 1.36–3.27; p linear = 0.0009) in participants without prevalent CHD (n = 2140 (including 140 HF cases). Accordingly, for the second to the fourth quartile of omentin-1 in comparison to the first quartile (reference) the present study observed HRs (95%-CI) of 1.40 (0.74–2.63), 1.67 (0.87–3.18), 2.16 (1.19–3.93), respectively (p linear trend = 0.008). In contrast, in participants with prevalent CHD at baseline a U-shaped association between omentin-1 and HF risk was observed (n = 240 (including 72 HF cases), p non-linear = 0.006, Fig. 1) with the following HRs (95%-CI) 1. Quartile: 4.94 (1.76–13.9), 2. Quartile: 2.49 (0.89–7.13), 3. Quartile: reference, 4. Quartile: 1.48 (0.59–3.68). No further interaction in the overall study population (Model 4) was observed between omentin-1 and sex, (p for interaction = 0.46), waist circumference (p for interaction = 0.06), prevalent diabetes (p for interaction = 0.20), prevalent hypertension (for interaction = 0.53) or hsCRP (p for interaction = 0.41) with respect to HF.

### Sensitivity analyses

All sensitivity analyses have been conducted in the fully adjusted model 4. After exclusion of cases that occurred during the first 2 years of follow-up (n = 2335) from the analysis the associations between chemerin (HR per doubling chemerin: 4.89; 95%-CI: 2.49–9.61; p < 0.0001) and omentin-1 (HR per doubling omentin-1: 1.39; 95%-CI: 0.90–2.13; p = 0.14) concentrations and the risk of HF in the overall population remained unaltered. Also, results from stratified analyses did not considerably changed. Regarding omentin-1, the association was modified by prevalent CHD (p for interaction = 0.02), showing a linear association in participants without prevalent CHD (p linear = 0.003) and a U-shaped association in participants with prevalent CHD (p non-linear = 0.007). Moreover, also after excluding probable and possible HF cases (n = 2317) from the analysis the associations of both chemerin (HR per doubling chemerin: 3.95; 95%-CI: 1.82–8.58; p = 0.0005) and omentin-1 (HR per doubling omentin-1: 1.14; 95%-CI: 0.65–1.98; p = 0.65) and the risk of HF remained unchanged. Further adjustment for N-terminal pro-brain natriuretic peptide (NT-proBNP) (n = 1346) did not substantially alter the risk estimates as follows: chemerin was strongly associated with HF risk (HR per doubling chemerin: 5.36; 95%-CI: 2.52–11.4; p < 0.0001) and omentin-1 was not significantly related to HF in the overall study population (HR doubling omentin-1:1.24; 95%-CI: 0.80–1.93; p = 0.33). Also results from stratified analyses did not considerably change when further adjustment for NT-proBNP was considered. The association between omentin-1 and the risk of HF was modified by prevalent CHD (p for interaction = 0.004), showing that the shape of the association was linear in participants without prevalent CHD (p linear = 0.009) and U-shaped in participants with prevalent CHD (p non-linear = 0.0001).

## Discussion

In this prospective analysis, we observed a strongly increased risk of HF in participants with high circulating chemerin concentrations at baseline. Participants in the fourth quartile of chemerin had a more than fourfold higher HF risk. However, omentin-1 was not related to HF risk in the overall study population. Interestingly, the association between omentin-1 and risk of HF differed according to the presence of CHD. The shape of the association between omentin-1 and HF risk was linear in participants without prevalent CHD, whereas a U-shaped association between omentin-1 and HF incidence was suggested in participants with prevalent CHD.

To our knowledge, this is the first prospective study investigating potential associations between chemerin and risk of HF in apparently healthy middle aged men and women. So far, only few studies investigated the association between chemerin and different cardiovascular endpoints i.e. one small prospective study investigated the association between chemerin and cardiovascular endpoints in 495 patients undergoing coronary angiography for the evaluation of established or suspected stable CAD, showing that patients with high chemerin concentrations were more often affected by cardiovascular events, defined by vascular deaths, non-fatal myocardial infarctions, non-fatal strokes, and the necessity of cardiovascular intervention^15^. Moreover it has been demonstrated that the expressions of chemerin mRNA and protein was higher in epicardial adipose tissue from patients with CAD^22^. Further, recent studies have shown that circulating serum chemerin concentrations were associated with the presence of CAD^8–12^ and severity of coronary atherosclerosis^8,9,12^. Despite that the mechanisms of chemerin and cardiovascular pathologies have not been investigated thoroughly so far^23^, a recent study by Rodríguez-Penas et al. proposed possible chemerin-related pathways on viability in murine cardiomyocytes^7^. Observations of this study may provide possible explanation for the strong association between higher chemerin concentrations and HF risk in the present study. First, Rodríguez-Penas et al. observed that chemerin induced apoptosis directly in cultured cardiomyocytes in a dose- and time-dependent manner^7^. Second, it has been shown that chemerin partially suppresses AKT (protein kinase B) phosphorylation at Thr308, which has been linked to apoptosis^7^. Third, it has been demonstrated that chemerin also increased the activity of caspase-9, which could have a direct implication in cardiomyocyte apoptosis through the activation of apoptotic mediators of the AKT pathway^7^. Indeed, apoptosis plays a decisive role in the development of HF^7,24^. In humans, an apoptosis rate ranging from 0.12% to 0.70% in failing hearts has been reported^7,24^. Interestingly, this small level of apoptosis is considered sufficient to cause HF and even very low levels of apoptosis (23 myocytes/10^5^ nuclei) have been detected to induce dilated cardiomyopathy and HF^7,24^. Given the strong association between chemerin and HF risk in the present study, it is possible to speculate that chemerin might cause cardiomyocyte apoptosis leading to HF.

Regarding omentin-1, the present study is the first prospective study investigating associations with risk of HF in apparently healthy participants. Former studies investigating the association between omentin-1 and different clinical intermediate cardiovascular phenotypes observed conflicting results. A possible reason for the controversy might be explained by the fact that the suggested cardio-protective associations of omentin-1 were mainly based on small studies performed in participants with existing diseases and possibly unfavorable cardiometabolic risk profile^17,18,25^. We may hypothesize that the role of omentin-1 in cardiovascular health probably differs between participants with preexisting metabolic disease or unfavorable metabolic conditions compared to apparently healthy individuals. In line with that, we observed in the present study different shapes of associations of omentin-1 and risk of HF dependent on the absence or presence of prevalent CHD. In participants without prevalent CHD the HF risk rose linear with higher plasma omentin-1 concentrations. In contrast a U-shaped relationship between omentin-1 and HF risk was observed in participants with prevalent CHD. Our previous study investigating the association between omentin-1 and the risk of stroke performed in an apparently healthy population strengthen our hypothesis, by showing that stroke risk was generally stronger in metabolically healthier individuals compared to high cardiovascular risk groups^19^. In detail, the association between omentin-1 and the risk of stroke was stronger in participants with normal waist circumference, low concentrations of triglyceride and hsCRP, high adiponectin concentrations or absence of metabolic syndrome compared to those with increased waist circumference, high concentrations of triglycerides and hsCRP, low concentrations of adiponectin and the presence of metabolic syndrome, respectively^19^. Therefore, we suspect a complex molecular interplay between omentin-1 and metabolic conditions regarding cardiovascular health. Intense research and well-designed experiments are needed, addressing the biological cardiovascular processes of omentin-1 with the risk of different major cardiovascular endpoints under different metabolic conditions^19^. Competition for potentially shared signaling pathways with different signaling efficiencies would in principle agree with our observations^19^.

Strengths of the current study include the prospective study design with high follow-up response rate and the rigorous case validation, and the availability of high quality data as a result of the standardized procedures enabling us to adjust for a large variety of potential confounders. Nevertheless, the present results are limited to middle aged Caucasian participants and might not be generalizable to other populations with different ethnic or age composition. Furthermore, the present findings are based on single measurement of chemerin and omentin-1 concentrations. However, in prior analyses we observed high reliability over time for chemerin^26^ and omentin-1^27^, suggesting that a single measurement may provide reliable risk estimates.

In conclusion, we observed that high plasma chemerin concentrations were associated with a higher risk of HF. Moreover, the association between omentin-1 and HF risk were modified by prevalent coronary heart disease, showing that the shape of association was linear in absence of prevalent CHD and U-shaped in participants with pre-existing CHD.

## Materials and Methods

### Study population

The European Prospective Investigation into Cancer and Nutrition (EPIC)-Potsdam study consists of 16644 women and 10904 men recruited between 1994 and 1998 from the general population in Potsdam and surroundings^28^. During study follow-up, information about incident diseases and other lifestyle factors was collected every 2–3 years^29^. The study was conducted in accordance with the Declaration of Helsinki. Participants gave their written informed consent, and study procedures were approved by the Ethics Committee of the Medical Association of the State of Brandenburg.

**Figure 2:**
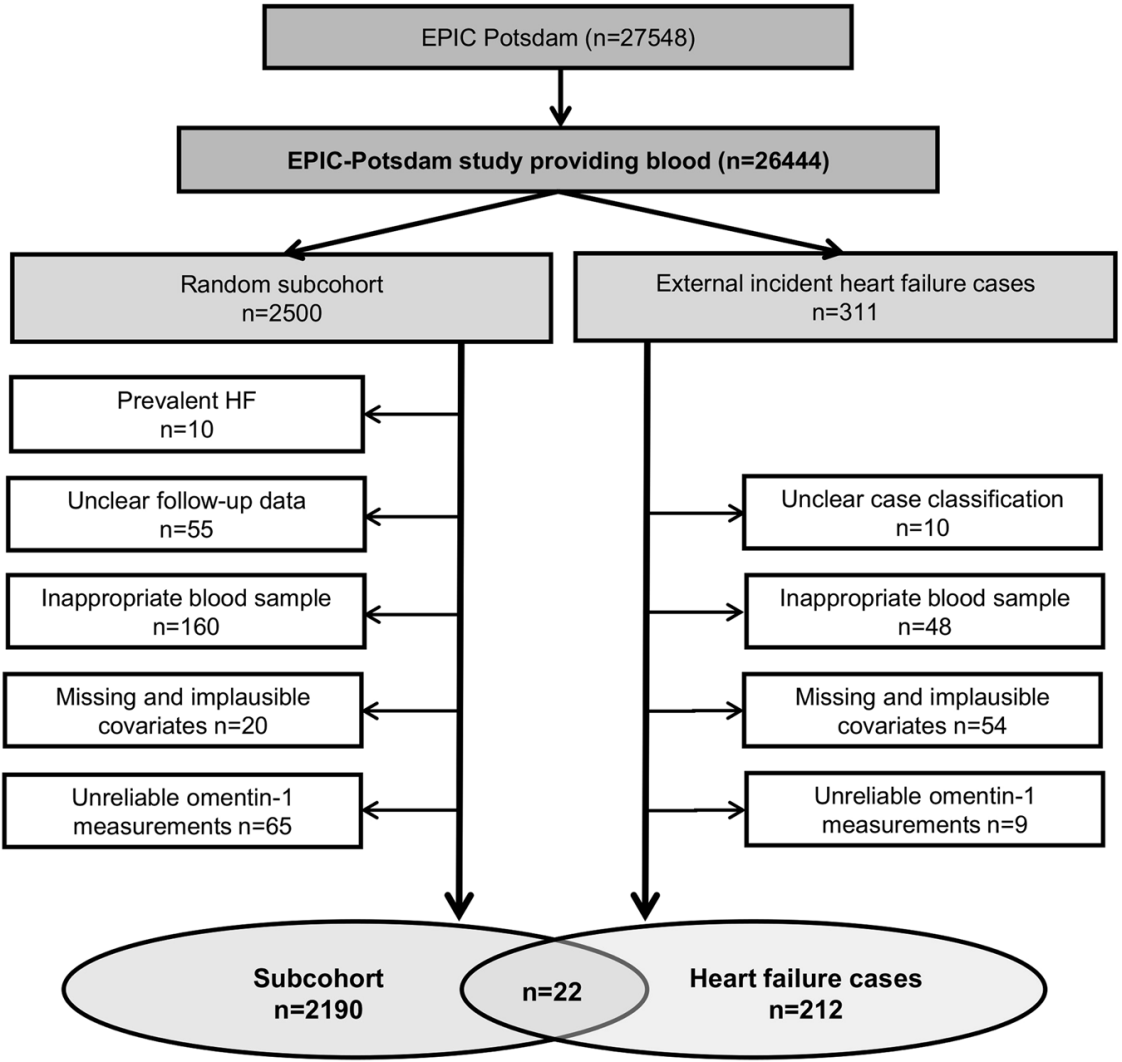
Flow diagram. Flow diagram for the exclusion criteria indicating the number of participants excluded from the present study.

A case-cohort study design was applied, using all incident HF cases and a subcohort of 2500 individuals randomly drawn from all participants of the EPIC-Potsdam study, who provided blood samples (n = 26444). This type of study design enables efficient analyses according to time and costs, whereas the results are generalizable in the entire cohort^30^. After exclusion of individuals with prevalent HF (n = 10), missing follow-up data (n = 55), inappropriate blood sample (n = 208), missing covariates (n = 74) and unreliable omentin-1 measurements (n = 74), the final study population consisted of 212 verified incident HF cases that occurred during 8.2 ± 1.5 years of follow-up and a subcohort of 2190 participants (overlap: 22 HF cases) (Fig. 2).

### Ascertainment of heart failure

HF cases were identified by self-report, death certificates (diagnosis I50 of International Classification of Diseases, 10th revision), and linkage to the hospital information system of the major hospital in the Potsdam area^31,32^. Additionally, participants with incident myocardial infarction or reported drug used typical for HF treatment have been actively enquired for HF^31,32^. Potential cases were validated by attending physicians using medical specific validation form following an established protocol. Confirmed cases were classified according to the Guidelines of the European Society of Cardiology (ESC-Guidelines) into definite (n = 149), probable (n = 52) and possible HF cases (n = 11)^33^. Cases were defined as ‘definite’ if two required criteria were fulfilled, i.e. presence of typical symptoms and objective evidence of cardiac dysfunction by echocardiography. Cases were characterized as ‘probable’, either if they were not symptomatic but had pathological evidence from echocardiogram, or if they were symptomatic but confirmed by other objective evidence (cardiac catheter, electrocardiogram, chest x-ray). Cases with less provided information were defined as ‘possible’^31,32^. Only medically verified incident cases of HF were included.

### Assessment of exposure and covariates

At baseline, 30 ml of venous blood was taken from each participant, processed and stored in tanks of liquid nitrogen at −196 °C or deep freezers at −80 °C^28^. The concentrations of the adipokines, chemerin and omentin-1, were measured with a sandwich ELISA by Biovendor (Brno, Czech Republic) at the Institute for Clinical Chemistry and Pathobiochemistry, Otto-von-Guericke University Magdeburg (Magdeburg, Germany). Chemerin was measured with intra-assay coefficients of variation (CV) between 5.1% and 7.0%, inter-assay CVs between 6.9% and 8.3% and a lower limit of detection of 0.1 ng/ml. The intra- and inter-assay CV of omentin-1 ranged between 3.2% and 4.1% and between 4.4% and 4.8%, respectively (limit of detection: 0.5 ng/ml), according to the manufacturer. Concentrations of total cholesterol, HDL-cholesterol, triglycerides and hsCRP had been analyzed at the Department of Internal Medicine, University of Tübingen (Tübingen, Germany) and at the Stichting Huisartsen Laboratorium (Breda, The Netherlands). As described elsewhere, NTproBNP was measured at the Institute of Clinical Chemistry and Pathobiochemistry, Otto-von-Guericke University Magdeburg (Magdeburg, Germany) using a solid-phase, two-site chemiluminescent immunometric assay (IMMULITE 2000 Systems Analyzers, Siemens)^32^. Concentrations below the lower limit of detection (20 pg/ml) were set to 10 pg/ml^32^. At baseline, trained personnel took the anthropometric measurements (weight, height) with participants wearing light underwear and no shoes with a precision of 0.1 kg and 0.1 cm, respectively. Waist circumference was measured at the midway between the lower ribs and the iliac crest and hip circumference over the buttocks. Lifestyle characteristics, including physical exercise, smoking history or education were documented at baseline by trained interviewers during a computer-assisted interview^28^. History of prevalent hypertension was defined as systolic blood pressure >140 mmHg or diastolic blood pressure >90 mmHg or self-reporting of a diagnosis or use of antihypertensive medication. The prevalence of diabetes at baseline was assessed by using information on self-reported diagnosis, medication records and dieting behavior. Dietary habits including alcohol consumption were assessed by a validated food frequency questionnaire. Participants were classified as having prevalent CHD, if they had either myocardial infarction or angina pectoris self-reported prior to recruitment.

### Statistical analysis

As depicted in Fig. 2, complete-case analyses were performed using only participants with complete data. Analysis of covariance (ANCOVA), adjusted for age and sex, was performed to investigate associations between chemerin, omentin-1 concentrations and several cardiovascular risk factors across quartiles of chemerin and omentin-1 concentrations within the subcohort. Variables were expressed as adjusted percentage or mean and 95%-confidence intervals (95%-CI). Correlations between the adipokines chemerin, omentin-1 and general and biochemical characteristics were assessed using Spearman sex- and age-adjusted partial correlation coefficients.

The associations between chemerin, omentin-1 and risk of HF were investigated by calculating hazard ratios (HR) and 95%-CI, modified for the case–cohort design according to the Prentice method. Age was used as the underlying time variable with entry time defined as the participant’s age at recruitment and exit time defined as the age at time of HF diagnosis or censoring. The final multivariable adjusted models included the following covariates: age, sex (Model 1), waist circumference, physical activity, education, smoking, alcohol consumption (Model 2), prevalent hypertension, diabetes, CHD (Model 3), and HDL-cholesterol, total cholesterol, triglycerides, hsCRP (Model 4). Concentration of triglycerides and hsCRP were natural log transformed. For the investigation of linear relationships (on a continuous scale) omentin-1 and chemerin were base 2 logarithm transformed, enabling the interpretation of increasing HF risk per doubling of values. Further, risk of HF was evaluated according to quartiles of the adipokines. The shape of the associations between chemerin, omentin-1 and HF risk in the entire sample and in subgroups was determined using restricted cubic spline Cox regression analysis adapted to the case-cohort design using five knots at the 5^th^, 35^th^, 50^th^ (reference), 65^th^ and 95^th^ percentile of chemerin and omentin-1 in the fully adjusted model. The Wald chi-square test was used to evaluate whether a nonlinear term of log2-omentin-1 added significant information to the model.

Possible effect modifications between important risk factors for HF (dichotomous) i.e. sex (male/female), obesity (waist circumference women ≤/>88 cm; men ≤/> 102 cm), inflammation (hsCRP </≥1.0 mg/l) and prevalent diabetes (yes/no), prevalent hypertension (yes/no), prevalent CHD (yes/no), and omentin-1 (continuous) or chemerin (continuous) in relation to HF risk were tested with cross-product terms in the fully adjusted model 4.

Several sensitivity analyses were carried out. We excluded the first 2 years of follow-up (n = 45) to account for the latency period between pathology and clinical diagnosis. Moreover, we excluded all probable and possible cases (n = 63). Because NT-proBNP has been proposed as an important marker for HF, additional analysis was performed with further adjustment of NT-proBNP, using a subsample of 1346 participants with NT-proBNP measurements (non-cases = 1141; cases = 205). NT-proBNP was natural log transformed.

The proportional hazard assumption was explored within the subcohort by plotting Schönfeld residuals for omentin-1 and chemerin against time. No violation was observed. A p-value < 0.05 was considered to be statistically significant. All statistical analyses were performed using SAS software, version 9.4 (SAS institute, Cary, N.C., USA).

### Data Availability

The datasets generated during and/or analyzed during the current study are not publicly available due to provisions of the written informed consent.

## Electronic supplementary material

Supplementary Information

## References

[1] Heart Failure Society of America *et al*. HFSA 2010 Comprehensive Heart Failure Practice Guideline. *Journal of cardiac failure* **16**, e1–194, 10.1016/j.cardfail.2010.04.004 (2010). 10.1016/j.cardfail.2005.11.005 16500560

[2] Bui AL, Horwich TB, Fonarow GC (2011). Epidemiology and risk profile of heart failure. Nature reviews. Cardiology.

[3] He J (2001). Risk factors for congestive heart failure in US men and women: NHANES I epidemiologic follow-up study. Archives of internal medicine.

[4] Romacho T, Elsen M, Rohrborn D, Eckel J (2014). Adipose tissue and its role in organ crosstalk. Acta Physiol (Oxf).

[5] Bluher M, Mantzoros CS (2015). From leptin to other adipokines in health and disease: facts and expectations at the beginning of the 21st century. Metabolism.

[6] Nakamura K, Fuster JJ, Walsh K (2014). Adipokines: a link between obesity and cardiovascular disease. J Cardiol.

[7] Rodriguez-Penas D (2015). The Adipokine Chemerin Induces Apoptosis in Cardiomyocytes. Cellular physiology and biochemistry: international journal of experimental cellular physiology, biochemistry, and pharmacology.

[8] Yan Q (2012). The association of serum chemerin level with risk of coronary artery disease in Chinese adults. Endocrine.

[9] Xiaotao L, Xiaoxia Z, Yue X, Liye W (2012). Serum chemerin levels are associated with the presence and extent of coronary artery disease. Coronary artery disease.

[10] Dong B, Ji W, Zhang Y (2011). Elevated serum chemerin levels are associated with the presence of coronary artery disease in patients with metabolic syndrome. Internal medicine.

[11] Lin X (2012). Elevated serum chemerin levels are associated with the presence of coronary artery disease in patients with type 2 diabetes. Clinical laboratory.

[12] Aksan G (2014). Association of serum chemerin levels with the severity of coronary artery disease in patients with metabolic syndrome. International journal of clinical and experimental medicine.

[13] Yoo, H. J. *et al*. Circulating chemerin level is independently correlated with arterial stiffness. *Journal of atherosclerosis and thrombosis* **19**, 59–66; discussion 67–58 (2012). 10.5551/jat.9647 22104178

[14] Zhao D, Bi G, Feng J, Huang R, Chen X (2015). Association of Serum Chemerin Levels with Acute Ischemic Stroke and Carotid Artery Atherosclerosis in a Chinese Population. Medical science monitor: international medical journal of experimental and clinical research.

[15] Leiherer A (2016). High plasma chemerin is associated with renal dysfunction and predictive for cardiovascular events - Insights from phenotype and genotype characterization. Vascular pharmacology.

[16] Cheng X (2016). Elucidating the pathophysiological significance of circulating omentin levels: Is higher better?. Atherosclerosis.

[17] Liu R, Wang X, Bu P (2011). Omentin-1 is associated with carotid atherosclerosis in patients with metabolic syndrome. Diabetes research and clinical practice.

[18] Greulich S (2013). Cardioprotective properties of omentin-1 in type 2 diabetes: evidence from clinical and *in vitro* studies. PloS one.

[19] Menzel J (2016). Omentin-1 and risk of myocardial infarction and stroke: Results from the EPIC-Potsdam cohort study. Atherosclerosis.

[20] Saely CH (2016). High plasma omentin predicts cardiovascular events independently from the presence and extent of angiographically determined atherosclerosis. Atherosclerosis.

[21] Jiang H (2017). Association between omentin and echo parameters in patients with chronic heart failure. Minerva cardioangiologica.

[22] Gao X (2011). Association of chemerin mRNA expression in human epicardial adipose tissue with coronary atherosclerosis. Cardiovascular diabetology.

[23] Bonomini M, Pandolfi A (2016). Chemerin in renal dysfunction and cardiovascular disease. Vascular pharmacology.

[24] Pillai VB, Sundaresan NR, Gupta MP (2014). Regulation of Akt signaling by sirtuins: its implication in cardiac hypertrophy and aging. Circulation research.

[25] Yoo HJ (2011). Association of circulating omentin-1 level with arterial stiffness and carotid plaque in type 2 diabetes. Cardiovascular diabetology.

[26] Eichelmann F (2017). Methodological utility of chemerin as a novel biomarker of immunity and metabolism. Endocr Connect.

[27] Wittenbecher C (2015). Reproducibility of Retinol Binding Protein 4 and Omentin-1 Measurements over a Four Months Period: A Reliability Study in a Cohort of 207 Apparently Healthy Participants. PloS one.

[28] Boeing, H., Wahrendorf, J. & Becker, N. EPIC-Germany-A source for studies into diet and risk of chronic diseases. European Investigation into Cancer and Nutrition. *Annals of nutrition & metabolism* **43**, 195–204, doi:12786 (1999). 10.1159/000012786 10592368

[29] Bergmann, M. M., Bussas, U. & Boeing, H. Follow-up procedures in EPIC-Germany–data quality aspects. European Prospective Investigation into Cancer and Nutrition. *Annals of nutrition & metabolism* **43**, 225–234, doi:12789 (1999). 10.1159/000012789 10592371

[30] Prentice RL (1986). A case-cohort design for epidemiologic cohort studies and disease prevention trials. Biometrika.

[31] di Giuseppe R (2017). Plasma osteoprotegerin, its correlates, and risk of heart failure: a prospective cohort study. European journal of epidemiology.

[32] Wirth J (2014). Relationship between N-terminal pro-brain natriuretic peptide, obesity and the risk of heart failure in middle-aged German adults. PloS one.

[33] Swedberg K (2005). Guidelines for the Diagnosis and Treatment of Chronic Heart Failure: executive summary (update 2005). Revista espanola de cardiologia.

