# Supplementary material for: Association between chemerin, omentin-1 and risk of heart failure in the population-based EPIC-Potsdam study
Source: Sci Rep. 2017 Oct 26;7:14171. doi: 10.1038/s41598-017-14518-2 (PMC5658383; doi:10.1038/s41598-017-14518-2)
Supplement: Supplementary file 1 — Supplementary Information [file 41598_2017_14518_MOESM1_ESM.pdf]

Association between chemerin, omentin-1 and risk of heart failure in the population-based EPIC-Potsdam study

Juliane Menzel, Romina di Giuseppe, Ronald Biemann, Clemens Wittenbecher, Krasimira Aleksandrova, Fabian Eichelmann, Andreas Fritsche, Matthias B. Schulze, Heiner Boeing, Berend Isermann, Cornelia Weikert

Supplementary Table S1. Partial spearman correlations between chemerin, omentin-1 and General and biochemical characteristics in the subcohort (n=2190)

	**Chemerin**	**Omentin-1**
Waist circumference [cm]	*0.40 (<0.0001)*	*-0.16 (<0.0001)*
Physical activity [h/week]	*-0.07 (0.005)*	*0.06 (0.005)*
Total cholesterol [mmol/l]	*0.15 (<0.0001)*	*0.04 (0.09)*
HDL-cholesterol [mmol/l]	*-0.19 (<0.0001)*	*0.16 (<0.0001)*
Triglyceride [mmol/l]	*-0.38 (<0.0001)*	*-0.09 (<0.0001)*
hsCRP [mg/l]	*-0.43 (<0.0001)*	*-0.10 (<0.0001)*
Adiponectin [µg/ml] [a]	*-0.20 (<0.0001)*	*0.22 (<0.0001)*
Chemerin [ng/ml]		*-0.04 (0.05)*
Omentin-1 [ng/ml]	*-0.04 (0.05)*	

Expressed as ρ (p-value), adjusted for sex and age.
[a] n=2153

Supplementary Table S2. Clinical characteristics of heart failure cases in EPIC-Potsdam according to ESC-Guidelines (definite, probable and possible heart failure cases)

		ESC-Guidelines diagnostic category		
	All cases	Definite	Probable	Possible
n (%)	212	149 (70)	52 (25)	11 (5)
Clinical symptoms n (%)	186 (88)	149 (100)	36 (69)	1 (9)
**Pathological findings n (%)**				
Echocardiogram	165 (78)	149 (100)	16 (31)	0
Cardiac catheterization	102 (48)	85 (57)	15 (29)	2 (18)
Chest x-ray	111 (52)	80 (54)	31 (60)	0
Electrocardiogram	127 (60)	96 (64)	30 (58)	1 (9)
**NYHA classification n (%)**				
I	27 (13)	13 (9)	12 (23)	2 (18)
II	65 (31)	56 (38)	9 (17)	0
III	42 (20)	33 (22)	9 (17)	0
IV	12 (6)	10 (7)	2 (4)	0
Unknown/ missing information	66 (31)	37 (25)	20 (38)	9 (82)
**Type of HF n (%)**				
Right-sided heart failure	9 (4)	4 (3)	4 (8)	1 (9)
Left-sided heart failure	147 (69)	114 (77)	31 (60)	2 (18)
Global heart failure	32 (15)	24 (16)	8 (15)	0
Unknown/ missing information	24 (11)	7 (5)	9 (17)	8 (73)
**Function of HF n (%)**				
Diastolic	12 (6)	9 (6)	3 (6)	0
Systolic	83 (39)	70 (47)	13 (25)	0
Both	43 (20)	32 (21)	9 (17)	2 (18)
Unknown/ missing information	74 (35)	38 (26)	27 (52)	9 (82)

Supplementary Table S3. Hazard ratios of HF according to quartiles of omentin-1

	Quartiles of omentin-1 levels					Per doubling of omentin-1	
	**Q1**	**Q2**	**Q3**	**Q4**	*p for trend*		*p-value*
Omentin-1 [ng/ml] [a]	286.5 (250.6-308.9)	365.4 (346.3-381.6)	441.8 (422.2-465.2)	574.2 (523.0-643.5)			
Subcohort participants (n)	547	549	548	546			
Heart failure cases (n)	32	44	52	84			
Model 1 [b]	Reference	1.26 (0.76-2.06)	1.09 (0.66-1.78)	1.42 (0.89-2.26)	*0.15*	1.40 (0.94-2.08)	*0.10*
Model 2 [c]	Reference	1.32 (0.79-2.21)	1.13 (0.68-1.89)	1.64 (1.01-2.65)	*0.05*	1.56 (1.03-2.36)	*0.04*
Model 3 [d]	Reference	1.26 (0.74-2.14)	1.08 (0.64-1.81)	1.41 (0.86-2.31)	*0.20*	1.38 (0.90-2.11)	*0.14*
Model 4 [e]	Reference	1.08 (0.64-1.82)	0.92 (0.53-1.59)	1.21 (0.72-2.02)	*0.41*	1.25 (0.80-1.96)	*0.32*

Hazard ratios and 95%-CI were derived from Cox proportional hazard regression.
[a] Quartiles are based on the distribution of omentin-1 within the subcohort expressed as median and interquartile range.
[b] Model 1: adjusted for age and sex.
[c] Model 2: additionally adjusted for waist circumference, physical activity, education, smoking, alcohol consumption.
[d] Model 3: additionally adjusted prevalent hypertension, diabetes, CHD.
[e] Model 4: additionally adjusted HDL-cholesterol, total cholesterol, triglycerides, hsCRP.
